# Seismic signature of the deadly snow avalanche of January 18, 2017, at Rigopiano (Italy)

**DOI:** 10.1038/s41598-020-75368-z

**Published:** 2020-10-29

**Authors:** Thomas Braun, Barbara Frigo, Bernardino Chiaia, Perry Bartelt, Daniela Famiani, Joachim Wassermann

**Affiliations:** 1 Istituto Nazionale di Geofisica e Vulcanologia, Roma, Italy; 2 Department of Structural, Building and Geotechnical Engineering, Politecnico di Torino, Torino, Italy; 3 WSL Institute for Snow and Avalanche Research SLF, Davos, Switzerland; 4 Geophysical Observatory, Munich University, Fürstenfeldbruck, Germany

**Keywords:** Environmental sciences, Natural hazards

## Abstract

Most snow avalanches occur unobserved, which becomes particularly dramatic when human lives are involved. Seismological observations can be helpful to unravel time and dynamics of unseen events, like the deadly avalanche of January 18, 2017, that hit a Resort-hotel at Rigopiano in the Abruzzi (Italy). Particle motion analysis and spectrograms from data recorded by a close seismic broadband station, calculation of synthetic seismograms, as well as simulation of the flow, allowed us to construct the dynamics of the snow avalanche that buried alive 40 people, killing 29. Due to the bad weather conditions, no visual observation was made, thus making it impossible to determine the exact moment of the avalanche and to report necessary observations of the dramatic event. On-site inspections revealed that the hotel was horizontally cut by shear forces and dislocated by 48 m in 70°N direction, once the increasing avalanche pressure exceeded the structural shear strength of the building. Within an eligible 24 min time range of the avalanche, we found three weak seismic transients, starting at 15:42:38 UTC, recorded by the nearest operating station GIGS located in the Gran Sasso underground laboratory approximately 17 km away. Particle motion analysis of the strongest seismic avalanche signal, as well as of the synthetic seismograms match best when assuming a single force seismic source, attacking in direction of 120°N. Simulation of the avalanche dynamics—calculated by using a 2D rapid mass movement simulator—indicates that the seismic signals were rather generated as the avalanche flowed through a narrow and twisting canyon directly above the hotel. Once the avalanche enters the canyon it is travelling at maximum velocity (37 m/s) and is twice strongly deflected by the rock sidewalls. These impacts created a distinct linearly polarized seismic “avalanche transient”s that can be used to time the destruction of the hotel. Our results demonstrate that seismic recordings combined with simulations of mass movements are indispensable to remotely monitor snow avalanches.

## Introduction

Seismology provides useful tools that can help to better understand the dynamics of seismic events, different from earthquakes, as e.g. volcanic eruptions, rock falls or huge landslides^1–5^. There are only a few examples in literature where seismology was successfully used to study avalanches^6–10^, probably because the density of snow is up to ten times smaller, compared to debris, which results in a reduced ground coupling and in a smaller seismic signal amplitude. On January 18, 2017, in a remote location in the Abruzzo region (Central Italy), a deadly avalanche buried 40 people under the Resort-hotel “Rigopiano”. In a dramatic rescue operation 11 people could be recovered, while for another 29 persons there was no way to escape. The bad weather conditions with heavy snowfall closed the access road, isolating the Rigopiano location from the outside world. The reduced visibility prevented any eyewitness report of the avalanche, thus the exact moment, as well as the dynamics of this catastrophic event, are still not confirmed. We use seismic recordings and on-site inspection, combined with numerical modelling, to reconstruct the dynamics and to determine the exact moment of the deadly avalanche.

### Chronology

**Figure 1:**
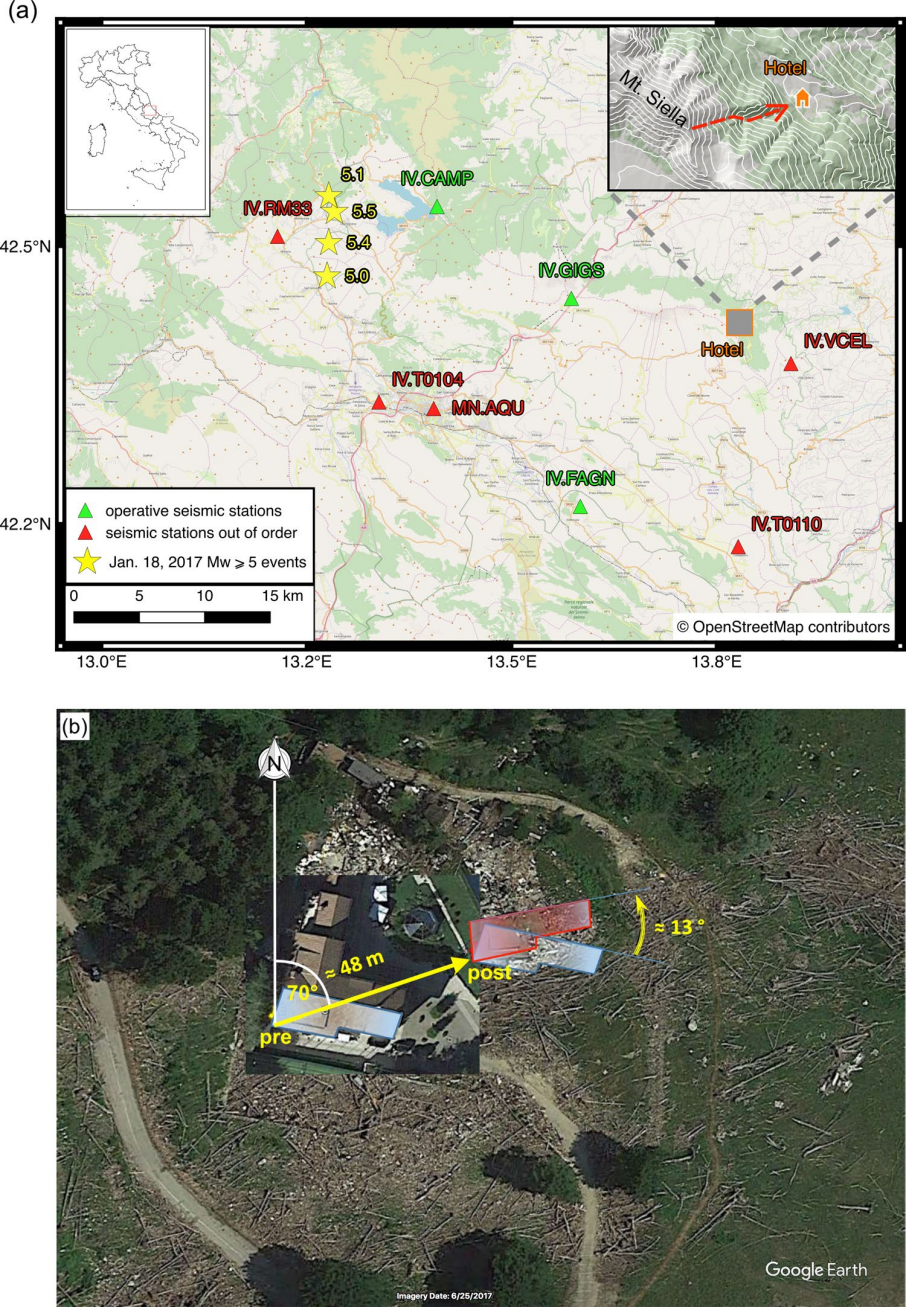
(**a**) Seismic stations of the INGV-network (triangles) installed in the Abruzzo area. The yellow stars indicate the earthquakes on January 18, 2017, which occurred at UTC 09:25:40 (M_w_5.1), 10:14:09 (M_w_ 5.5), 10:25:23 (M_w_ 5.4), and 13:33:36 (M_w_ 5.0). Only station GIGS, situated at 17 km W of Rigopiano, recorded a signal related to the avalanche that hit the hotel*.* (**b**) The avalanche caused a dislocation of the hotel’s upper floor by 48 m in ~ 70°N direction (measured by the dislocation from the SW-edge of the building from “pre” to “post”) and a ~ 13° anticlockwise rotation. [This figure has been constructed using QGIS^13^, https://www.qgis.org/en/site/; Map data copyrighted OpenStreetMap contributors; data is available under the Open Database License (https://www.openstreetmap.org/copyright), retrieved from https://planet.openstreetmap.org and Google Earth, imagery date 06/25/2017, https://www.earth.google.com].

A brief cold period lasting from January 15 to 19, 2017, caused abundant snowfall in the Marche and Abruzzo regions, reaching a snow depth of about 2 m at altitudes above 1000 m.a.s.l. in the Sibillini and Gran Sasso mountains. In the morning of January 21, 2017, and thus three days after the avalanche, the Meteo-service agency^11^ estimated a fresh snow depth of 2 m near hotel Rigopiano in the location of Farindola (1200 m.a.s.l., Pescara Province), and even more on top of Mt. Siella (2027 m.a.s.l., Fig. 1).

On January 18, 2017, between 09:25 and 13:33 UTC four seismic events of magnitude M ≥ 5 occurred at a distance of circa 45 km W off the location of hotel Rigopiano (yellow stars in Fig. 1a) causing tremors perceptible as far as Rome and Naples. As those earthquakes were distinctly felt also at Rigopiano, spreading panic among the hotel residents, the question arose, whether the avalanche could have been seismically triggered^12^. Given the large epicentral distance and a minimum 2 h time offset between the latest M5 event and the snow mass detachment, we consider it as very unlikely that the avalanche was released by ground oscillations from those events, while temperature increase in the course of the day may, however, play an important role for triggering the avalanche.

Due to the bad weather conditions, fresh snow interrupted the 9 km long access road to the hotel, cutting also power and telecommunication lines. Therefore, none of the entrapped 11 employees and 29 guests had any chance, neither to receive help from the outside, nor to leave the hotel. In the afternoon of January 18, 2017, almost two hours after the last M5.0 earthquake of 13:33 UTC (all times are indicated as UTC = local time − 1 h), a snow avalanche detached from Mt. Siella at 1969 m.a.s.l., increasing its mass, while running down the 2 km long wooded valley heading for Rigopiano. The power of its masses buried the Resort hotel with 40 residents and swept away the entire upper floors comprising the roof.

There are only two indirect eyewitnesses of this deadly event: One is the hotel maintenance technician (F.S.), who experienced the avalanche inside the hotel’s heating room, a small technical compartment, which was indeed concerned by entering snow, but did not collapse. F.S. freed himself, after approximately 15 min; he later reported: “the avalanche was silent […]. No roar, no air movement. A loud rustling”^14^. The second eyewitness is the hotel guest (G.P.) who, shortly before the avalanche, went outside to the parking area to pick up something in his car. On his way back to the hotel, he heard noises and squeaks and was partially submerged by the snow. Later, he said: ”I saw the mountain falling on the hotel”^15^. It was G.P. who transmitted the first emergency call at 16:40 UTC. Any earlier attempt to call for help was unsuccessful due to the intermittent mobile phone connection, leaving unanswered the question about the exact moment of the avalanche.

To define an accurate timing of the avalanche is of great importance for the victims’ relatives, as well as for issues regarding the rescue operations. The last phone call from hotel Rigopiano before the avalanche was taken at 15:30, and as reported by BBC^16^, the avalanche struck sometime before 16:40, when the first emergency call was received. As reported by the newspaper “La Repubblica”^17^, subsequent inspections of the victims’ mobile phones revealed that on 16:09 one of the guests sent an audio WhatsApp Message (WAM) to the sanitary emergency of the Province capital Chieti, with the words: “Hotel Rigopiano, collapse, avalanche, survivors, missing”. This information shortens the time window of the possible avalanche from 70 to 39 min. A further WAM “Help, I’m blocked by the rubble”, written at 15:54 but never sent, restricts the avalanche time window to 24 min, starting at 15:30.

### Avalanche parameters retrieved from on-site inspections

The track and the trajectory of the Rigopiano avalanche are easy to identify from on-site observations. The rapid flow of avalanches hits a corridor in the beech forest, carving parabolic curves, and impacts the hotel by spreading branches of trees, cars, lanterns, boulders and roots. Several on-site inspections revealed that, from a dynamic point of view, the event of January 18, 2017, could be classified as “mixed” avalanche, i.e. the combination of a skimming flow and a powder part. Moreover, the dry snow of medium–high density with weak internal friction and high velocities led to a predominantly deforesting action. As tree breaking extracts little kinetic energy from the avalanche^18,19^, the forest did not decelerate the avalanche significantly. Large parts of the forest were thus entrained and transformed the avalanche in a sort of fast landslide of snow and wood, steamrolling the fragile parts of the forest and dragging the trunks. The avalanche increased its already high kinetic energy travelling the entire slope, building up its mass and varying its density caused by the entrainment of the fresh snowpack, rocks and the uprooted beech forest. The avalanche impacted the hotel by burst of compacted snow mixed with wood, dirt, rocks and boulders, growing the overall impact pressure of flow on the Hotel. The high kinetic energy of the avalanche due to its elevated density—from the entrainment of wood and trunks—resulted in an increased impact velocity when striking the hotel, dislocating the upper part (Fig. 1b). The Rigopiano event can be defined as a “wood-and-snow” avalanche: the fast snow flow broke the beeches already at the beginning of the sliding zone, crossing a rocky spur (usually bypassed by avalanches) and rising its destructiveness without any deceleration effect. At the run-out zone, the snow and wood deposits about 4 m thick were measured over a distance of nearly 450 m downstream from the hotel, where the slope of this grassy plateau does not exceed 4° (against horizontal). The total distance travelled by the dense avalanche reaches 2.3 km. The front of the avalanche instantly overwhelmed the hotel, destroyed the masonry walls, broke the reinforced concrete columns and made the beams/columns connections collapse, thus weakening the bearing structure.

The mass of snow and debris shifted the upper floors of the hotel by approximately 48 m downstream, rotated them slightly by 13° anticlockwise (Fig. 1b), teared them apart and entered the lower and underground floors of the hotel. This westernmost portion of the building constructed at the end of the 1960s along the upstream side was facing the frontal impact of the flow, with an angle of incidence approximately orthogonal (less than ± 20° with respect to the perpendicular) and (minimum) height of the second floor above ground.

According to the local topography, the upstream part of the hotel building resides in a recessed position with respect to the ground level covered by 3 m thick snowpack, protecting in this way the lower floors of the building against the approaching avalanche. The mass flow was thus deviated directly to the second floor, which collapsed subsequently by shearing, without affecting the foundation.

### Seismic data analyses

Due to the heavy snow falls in mid-January 2017, most of the—often solar-panel-powered—seismograph stations failed (red triangles in Fig. 1a). On January 18, 2017, only few seismic stations were running (green triangles in Fig. 1a); station GIGS was the closest operational station to hotel Rigopiano (distance of 17 km). Prior to the data analyses, we verified the horizontal orientation of the three-component seismic broadband sensor of station GIGS (Nanometrics Trillium 240 s), installed inside the Gran Sasso Tunnel system and found a misalignment by − 36°N to be considered in the following analyses.

**Figure 2:**
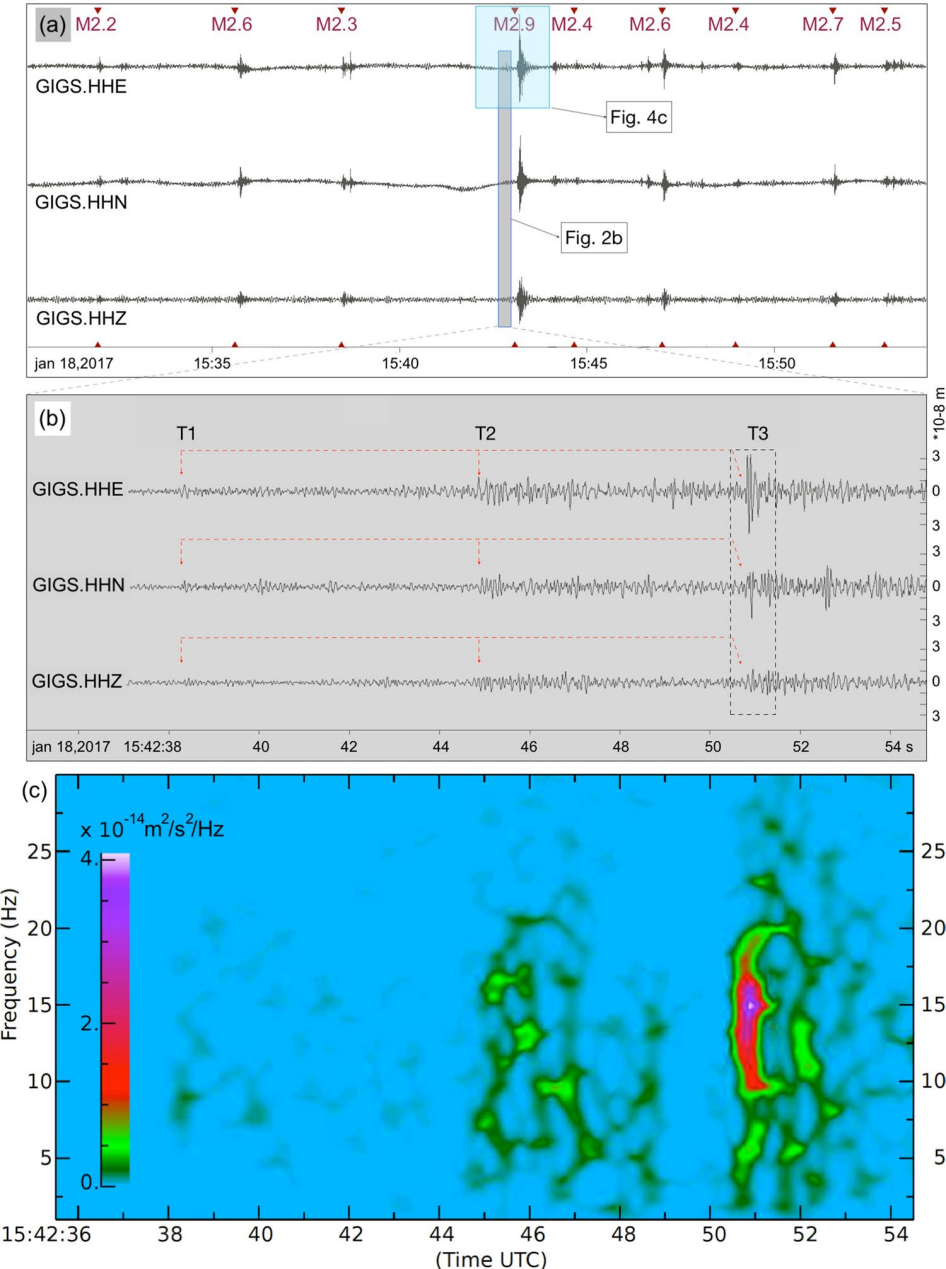
Seismic traces recorded at station GIGS: (**a**) 24 min zoom for the avalanche time window, (**b**) 20 s zoom, containing the displacement trace generated by the avalanche, (**c**) spectrogram of the E-component of station GIGS plotted for the 20 s-time window of (**b**), generated by the avalanche. [Panels a and b have been plotted by using Snuffler, https://pyrocko.org; Panel c was realized using SAC-software: https://ds.iris.edu/ds/nodes/dmc/forms/sac/].

With the aim to determine the precise moment of the avalanche, we first inspect the seismograms of the nearest seismograph stations of the INGV-network (Fig. 1a). Figure 2a shows the eligible 24 min avalanche time window for all three components of station GIGS, which was scanned for any “suspicious” signal that could have been generated by the avalanche. The recordings are obviously dominated by the continuous seismic activity of the Central Italy sequence (inverted red triangles in Fig. 2a), which complicates the search.

At 15:42:38 the seismic trace of station GIGS started to record small transients on all three components, without any coincident signals at the other running stations of the network (triangles in Fig. 1a), which indicates that they had not been generated by the Central Italy seismic sequence. The signal has a duration of approximately 15 s and is composed by three distinct onsets (called hereafter avalanche transients T1–T3). T1 (1st red arrow in Fig. 2b) weakly starts at 15:42:38, followed by an amplitude increase 7 s later (T2—2nd red arrow) and culminating in the very sharp high-frequency (~ 14 Hz) transient (T3—3rd arrow). T3 lasts for less than 0.5 s and is particularly evident on the horizontal-components, indicating an SH-wave. The peak ground velocity reaches a value of 3.2 × 10^−6^ m/s, and a corresponding peak ground displacement of 3·10^−8^ m. The spectrogram in Fig. 2c shows the three distinct patterns as spectral energy in the frequency band of 1–20 Hz, with continuously increasing seismic energy at 38 s (T1), 45 s (T2) and 51 s (T3), respectively, the latter showing a distinct maximum at ~ 14 Hz (violet).

**Figure 3:**
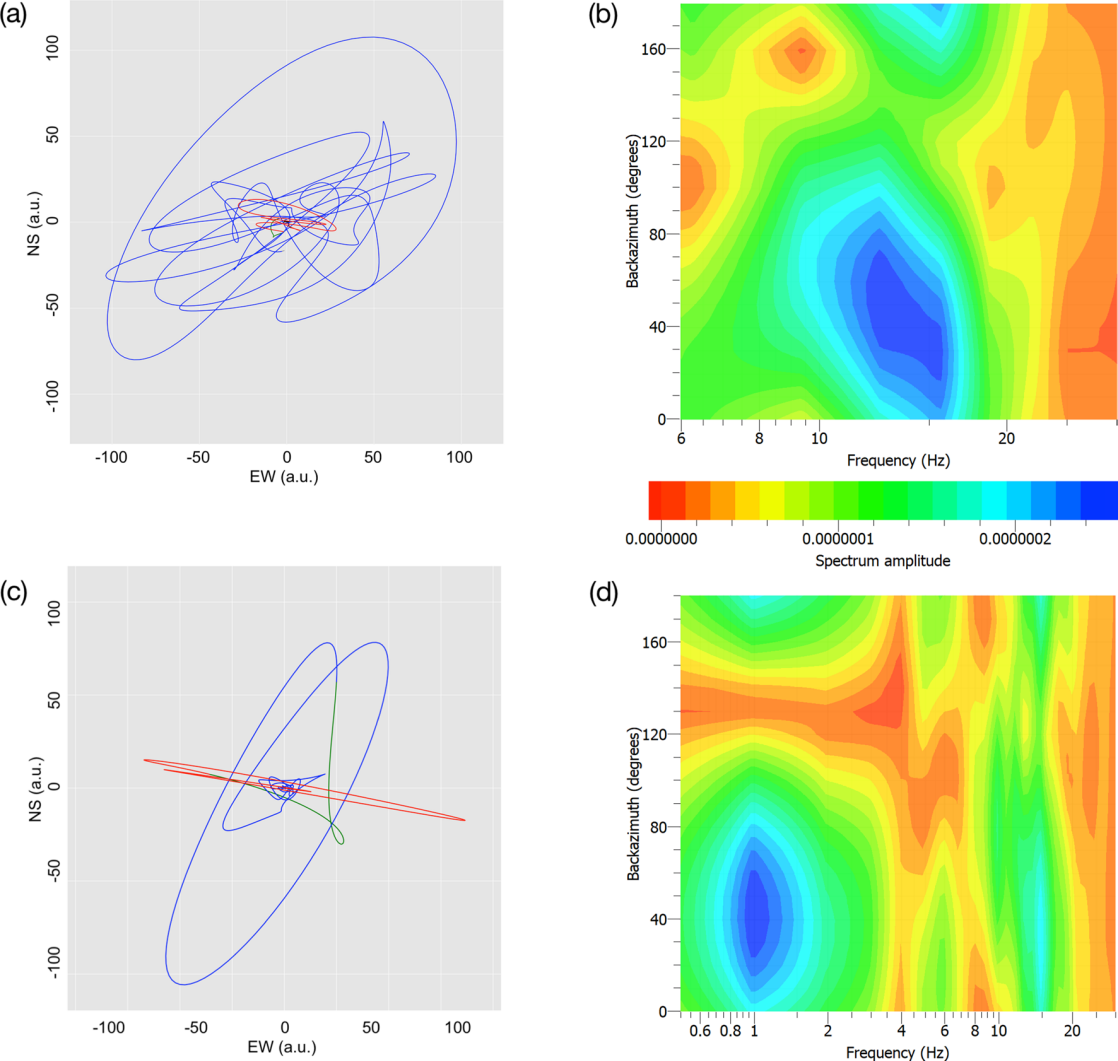
Directional analysis of the avalanche signal: (**a**) particle motion of the data and (**c**) of the synthetic seismograms (arbitrary units).Rotated frequency spectra calculated from combining the horizontal components of station GIGS for a time window of (**b**) 0.3 s and (**d**) 1.0 s around AT. [Panels b and d have been constructed by the software Geopsy, https://www.geopsy.org; Panels a and c have been realized using the software ObsPy https://github.com/obspy/obspy/wiki and SW4^20^ https://geodynamics.org//cig/software/github/sw4/v2.0/sw4-v2.0.tgz].

In a second step, we try to determine the direction(s) under which the avalanche transients reach station GIGS, but only T3 shows a signal-to-noise ratio high enough to be further examined. Figure 3a shows the particle motion of T3 plotted in the horizontal NE-plane). The initial (red) part of the seismograms points approximately in direction ~ 110° (P-wave), while the large amplitudes (blue) show a mean direction of ~ 45° and can be interpreted as SH-waves (T3).

To retrieve a statistically significant backazimuth of the short duration of T3, we compute the rotated frequency spectra of the horizontal components recorded at station GIGS, by calculating a series of Fourier spectra in the range from 0° to 180° (by steps of 10°) and present the outcome in the frequency-azimuth plane (Fig. 3b,d). Figure 3b shows the rotated frequency spectra for the 0.3 s long time window around T3. The maximum spectral energy results at a frequency of ~ 14 Hz and reaches station GIGS at a backazimuth of approximately 45°.

In a third step, we compute synthetic seismograms of the low frequency part of the T3 (0.8–3 Hz) and compare the corresponding particle motions with those of the band-pass filtered data (0.8–3 Hz) recorded at station GIGS (Fig. 3a). We use the software package SW4^20^, implementing a rough version of the true 3D topography (SRTM) and—according to the unidirectional movement of the avalanche—we assume as seismic source a shallow horizontally orientated single force (Gaussian shaped source time function in a frequency range of 0.8–3 Hz). Based on a velocity model computed for estimating the regional moment tensors of the aftershock data from the 2009 L’Aquila earthquake sequence^21^, we calculate the synthetic seismograms for station GIGS by varying the single force direction in azimuthal steps of 10°. The result is that the synthetics (Fig. 3c) fit the particle motion of the observed data (Fig. 3a) best, when assuming a single force pointing in direction 120°N. For the observed, as well as for the synthetic seismogram, both hodographs start with a linearly polarized P-phase, pointing to a backazimuth of 105°–110° for observed data (Fig. 3a) and in the 100°N direction for the synthetics (Fig. 3c), followed by an almost perpendicular polarized SH-wave.

As the frequency bands of the recorded seismic data (14 Hz for T3) and synthetic seismograms (0.8–3 Hz) differ substantially, the rotated frequency spectra have been repeated for an extended time window of 1 s around T3, revealing seismic energy around 1 Hz, which arrives from the same backazimuth of 45° as for the high-frequency (Fig. 3d).

Concluding, the 15 s-lasting seismic signal related to the avalanche, shows three distinct phases: at 15:42:38 (T1), 7 s later (T2), followed by the T3 another 6 s later. The SH-part of the T3 reaches station GIGS from 45°, which fits best to the particle motions of synthetic seismograms computed for a single force pointing in direction 120°N.

### Avalanche modelling

To better understand the dynamics of the Rigopiano avalanche, we simulated the event by using the extended version of Rapid Mass Movement Simulator (RAMMS^22^), able to compute a 2D simulation of the rapid movement’s dynamics on a 3D alpine terrain. RAMMS is able to calculate a continuous numerical model of a mixed avalanche composed by a denser—but nevertheless fluidized—part (the core) and a nubiform part (powder snow)^23^. RAMMS considered further the process of the entrainment (snowpack erosion, variability of density, temperature and humidity of the snow in motion along the path)^24^ and allowed to calculate for the two-avalanche components pressure, velocity, density and flow height for each point of the running slope. The complexity of the avalanche dynamics saw the flow initially running on the non-wooded open slope, continuing on a partially incised slope strongly interacting with the forest, becoming almost a wood-and-snow mixed flow^18,25^. The simulation provided an estimation of the most probabilistic dynamics of the Rigopiano avalanche event of January 18, 2017. According to^26^, the main inputs of the simulation are:

- the released slab is characterized by an average thickness of 2 m with a density of 250 kg/m^3^;
- the average inclination of the release area is 32°, from 1890 and 1760 m.a.s.l., with a total surface of about 38,500 m^2^, a released mass of 19,255 t and volume of 77,000 m^3^
- the final avalanche density is assumed to increase to 450 kg/m^3^ in the run-out area, resulting as an average between the typical value of snow in the avalanche flow plus incorporated wood of teared beeches.

Uncertainties in the avalanche calculations denote the variations from the average values of the initial parameters release height and slab density. In the fracture zone we employ an average release height of h = 2 m assuming a possible variation of ± 0.5 m. Concerning the average value of the fracture slab density ρ we state: the snow density of fresh snow is lower than the average (50 kg/m^3^ < ρ < 150 kg/m^3^), while the density of the older, settled snow is somewhat larger (ρ > 150 kg/m^3^). The average values (h = 2 m and ρ > 250 kg/m^3^) therefore provide good estimates of the overall release volume, which is necessary to make computationally tractable and realistic simulations.

**Figure 4:**
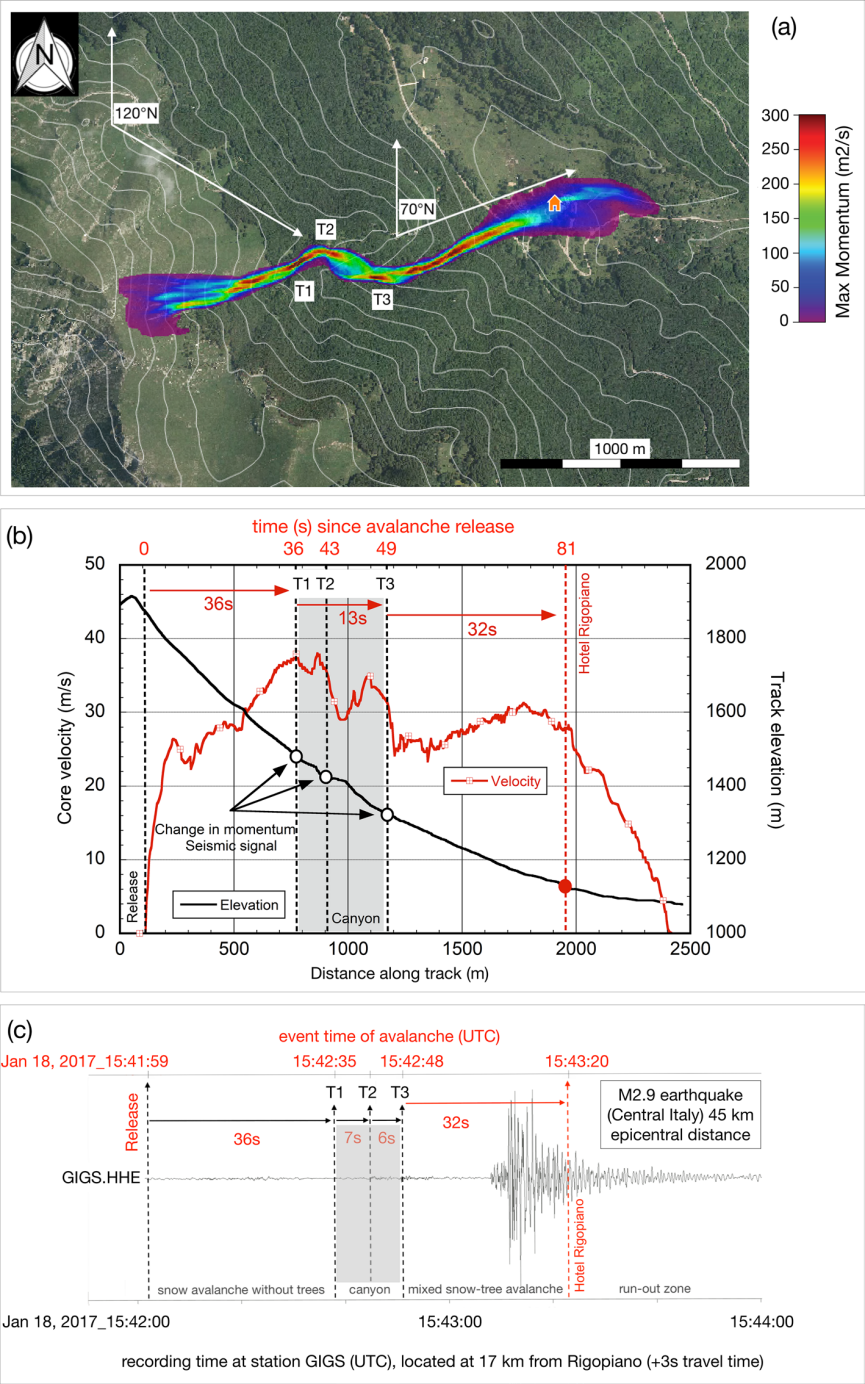
Track simulation of the Rigopiano avalanche of January 18, 2017, and comparison with the seismic signal: (**a**) progression of the modelled momentum along the avalanche track. At the entrance into the canyon (T1), and the deflection points (T2, T3) maximum momentum changes are expected. (**b**) Track elevation (black line), avalanche velocity (red line) and corresponding Time (s) after nucleation, as function of distance from the release area. (**c**) HHE-component of seismic recording (see Fig. 2a) indicating the onset times of the three avalanche transients (at 15:42:38, 45 and 51 UTC). The avalanche reaches the hotel after approximately 81 s inside the coda of a regional M2.9 event. [These figures have been realized using QGIS^13^, https://www.qgis.org/en/site/, representing results of RAMMS^22^, plotted, together with 50 m contour lines, on “2013 AGEA digital orthophoto” available by Regione Abruzzo WMS service, https://geoportale.regione.abruzzo.it/Cartanet/catalogo/cartografia-di-sfondo-raster/ortofoto-digitale-agea-2013].

The avalanche simulation revealed that the avalanche developed in approximately 2 min, reaching a high peak velocity of approximately 38 m/s (~ 136 km/h) along the track at about 1450 m.a.s.l. The trajectory of the Rigopiano avalanche can be divided into two distinct zones (Fig. 4a): (i) the upper avalanche track from 1900 to 1500 m.a.s.l., with a mean slope angle of 30° (against horizontal), is smooth, steep and free of trees. During its down-flow the avalanche accelerated and entrained fresh snow, entering a narrow canyon at 1500 m.a.s.l. (T1 in Fig. 4a), changing its channel width from 80 to 40 m. The canyon shape causes the avalanche to change direction twice, each time being deflected by an angle of approximately 45°. After entering the canyon, the avalanche slows to a mean speed of 35 m/s (Fig. 4a,b), reaching the first deflection point (T2) after 7 s. Since the deflection points are separated by a distance of 250 m (T2 and T3 in Fig. 4a), we estimate the second impact (deflection) to occur at about 6 s after the first. At the deflection points, where the avalanche changes abruptly its flow direction, we expect large impact forces on the canyon sidewalls and therefore the generation of a significantly energetic seismic signal. It takes about 13 s for the avalanche front to navigate the canyon, the entire avalanche, including the tail, requires an additional ~ 10 s to flow entirely through the narrow channel. The avalanche then departs the canyon, entering the lower, forested track segment, reaching the hotel after another 32 s. This segment starts at an elevation of 1300 m and points towards the hotel. Topography constrains the avalanche to flow through a dense beech forest, carving a well-defined destructive corridor. After leaving the canyon, the simulation results show the avalanche flowing directly towards the hotel, stopping at the road (1080 m.a.s.l., Figs. 1b, 4a).

The flowing part (core) of the avalanche hit the hotel with a speed of about 28 m/s (≈100 km/h) and at the simulation time of approximately 81 s ± 5 s, the maximum impact pressures reached 395 kPa with an avalanche density of about 400 kg/m^3^. After the initial peak (the arrival of the flowing front), a speed of 12 m/s is hold up for further 15 s with a corresponding decrease of the avalanche body and its impact pressure from 395 kPa to about 79 kPa. The avalanche entrained 103,000 m^3^ total volume of snow, obtaining a growth index of 2.3.

## **Discussion and conclusions**

We investigated a 15 s-lasting seismic signal, recorded at station GIGS (located approximately 17 km away from the avalanche) that occurred at approximately the same time as the catastrophic event at Rigopiano and that cannot be associated to any earthquake of the Central Apennines seismic sequence. Besides, the significant time span between the four M ≥ 5 events—which occurred between 09:25 and 13:33—and the snow avalanche event, makes the direct triggering effect unlikely.

On-site inspection clearly revealed that the avalanche caused a sudden horizontal shearing of the hotel building’s second floor, which collapsed after accumulation of the snow masses along the external wall of the ground floor redirecting the avalanche to the second floor collapsing subsequently by shearing without affecting the foundation and thus without an efficient coupling into the ground.

The question is, whether shearing and dislocation of the hotel building’s upper floor is capable to generate a seismic signal strong enough to be recorded by a seismic station located at a distance of 17 km? We are convinced that the answer is “No”.

To generate a seismic signal by an avalanche, a land slide or a rock fall, a strong force coupling between the mass flow and the ground is needed. Especially in the case of a snow avalanche, when the involved densities of the moving mass are relatively small, this coupling can arise either by the avalanche hammering onto the ground in the perpendicular direction of the flow, or when the avalanche impacts sidewalls, thus significantly changing its slope-parallel flow direction. As the slope-parallel velocities can reach 150 km/h, more seismic energy is generated by impacting sidewalls or buildings (obstacles). The main force of the avalanche is exerted downhill in the slope-parallel direction, while forces in the slope-perpendicular direction remain comparatively small. In fact, these forces are usually taken to be close to hydrostatic, and therefore depend on the height and density of the flowing snow. Sidewalls are thus ideal, because the large slope-parallel momentum of the avalanche is transferred directly into ground. When an avalanche flows on a smooth slope (without obstacles) almost no seismic energy couples to the ground, requiring seismic sensors to be installed in the near vicinity to measure any potential avalanche induced ground shaking.

Concluding, a significant seismic signal generated during the impact of the avalanche with a building can only be generated if the building can withstand the avalanche forces, transferring the momentum of the impact directly to the foundation sub-structure and then to the ground.

At Rigopiano the huge avalanche volume of 77,000 m^3^ is characterized by low mean densities of 250 kg/m^3^ (in the release zone and the forest-free upper part), increasing up to 450 kg/m^3^ (inside the mixed wood-and-snow avalanche that reaches the run out area), summing up to a total mass of 19,255 t. Rockfalls or landslides of similar volumes are characterized by much higher density values (factor 5–10), such that the normal force exerted couples seismic energy into the ground, generating the typical long-lasting seismic signal during the downhill movement^1,3,4^.

The seismic recordings show three distinct “avalanche transients” (T1,T2,T3 in Fig. 2b), which synchronize well with the main momentum changes revealed by the avalanche simulation (T1, T2, T3 in Fig. 4). We believe that the seismic signal is related to the change in momentum (avalanche height x avalanche velocity, Fig. 4a). Considering that on its way downwards, the avalanche mass increases continuously, the momentum of the moving mass flow changes significantly when its velocity changes due to narrowing of the flow channel (canyon) or deflection^9,10^. The bigger the change, the bigger the force.

This is confirmed by the directional analysis. The particle motion of the avalanche transient with the highest amplitude (T3) reveal a P-phase pointing away from GIGS in direction 105°–110°N, followed by an SH-wave in NE-SW direction. According to the kind of movement of an avalanche flow along a surface, we assume single forces as seismic sources. To reproduce the hodograph of the seismic signal recorded at station GIGS, we calculate synthetic seismograms for a single force varying the attack angle in steps of 10°. The corresponding particle motion diagrams of the synthetics fit the data best for the single force in direction of 120°N (SF120°N in Fig. 4a) concordant to the impact direction of the avalanche on the sidewalls of the canyon, rather than for 70°N (SF70°N in Fig. 4a) the direction the avalanche hits the hotel building. The seismic trace and the corresponding spectrogram (Fig. 2c) reveal two distinct earlier phases (at 15:42:38 s and 45 s) before the T3 (at 51 s). These phases show a similar spectral energy pattern as T3 and are temporally separated by two time intervals of 6–7 s, which are also found by the avalanche simulation passing through the canyon.

While GPS-timing of the seismic recordings and avalanche parameters retrieved from on-site inspections can be considered as “precise”, some words have to be said about uncertainties of the calculated and modeled parameters.

The horizontal orientation of the seismometer inside the Gran Sasso Tunnel was checked by a Fibre Optic Gyrocompass and revealed a misalignment of − 36°, which are thence considered in all calculations. We estimate the backazimuths uncertainties of the GIGS-traces as at least 10°, five times higher than the 2° aperture angle between the release area and the run-out zone of the avalanche (with respect to GIGS). In our argumentation the backazimuths of the seismic avalanche transients prove that the corresponding seismic source is located ESE of GIGS, and not in the north-west direction, as for the epicentres of the concomitant seismic sequence in the Central Apennines (Fig. 1a). Accordingly, also synthetic seismograms are calculated for single force pointing in direction in steps of 10°, which is precise enough to distinguish between the two single force directions of 70°N and 120°N, in favour of the latter solution.

Hundreds of avalanche models have been calculated, varying snowpack erosion, density, temperature and humidity of the snow in motion along the path. All simulations result in slightly different final values for pressure, mass, density, snow pack height and velocity. The only simulation parameters relevant for confirming the seismic signature of the Rigopiano avalanche, are, however, the momentum changes and the associated travel times, at the moment the avalanche passes the canyon (T1, T2, T3, Fig. 4). As the topography of the avalanche track is well known, it can be stated that the uncertainties of the relative travel times—when passing the short and narrow canyon (T1, T2, T3, in Fig. 4)—do not exceed ± 0.5 s, respectively.

Coming back to the question about the exact timing we compare the variation of the simulated track elevation and avalanche velocity (Fig. 4b) with the temporal evolution of the avalanche recorded by the seismogram (Fig. 4c). As the seismic recordings of events occurring at Rigopiano take about 3 s travel time to reach station GIGS 17 km away, Fig. 4c indicates two different time scales: in red, the Time (UTC) shifted by the travel time correction of 3 s and in black, the UTC-timing for the seismogram.

- 15:41:59 ± 2.5 s: avalanche release
- 15:42:35 ± 0.5 s: T1 is generated once the avalanche enters the canyon
- 15:42:42 ± 0.5 s: T2 first deflection
- 15:42:48 ± 0.5 s: T3 second deflection
- 15:43:20 ± 5.0 s: the avalanche reaches the hotel.

The large uncertainty in the definition of the release time (± 2.5 s) is associated with the break-up of the fracture slab into smaller fragments. This process determines the transition from the motion of a solid block to a granular fluid and therefore the initial speed of the avalanche. Once the granularization process is complete, the uncertainties in the model calculations decrease significantly to ± 1.0 s.

We calculated that the theoretical onset time of a hypothetical seismic signal caused by the impact of the avalanche with the hotel, has to be expected at ~ 81 s ± 5 s after the avalanche release. This instant falls exactly in the eligible time window when station GIGS recorded a M2.9 earthquake from the Central Italy seismic sequence, thus masking in its S-wave coda any hypothetical signal caused by the detachment of the hotel’s upper floor due to the avalanche (see Fig. 4c).

Between January 15 and 18, 2017, the cumulated height of fresh snow in 3 days at an altitude of 1880 m a.s.l. was close to 360 cm (without counting local snowdrift effects). The rising temperatures^27^ above 0 °C in the course of the day in combination with the growing snowpack may have contributed more significantly to the destabilization of the snow slope, leading to the stress causing the energetic triggering of the deadly avalanche^28,29^. As a matter of fact, between January 15 and 18, more than 520 large or very large avalanche events occurred in that area of the Italian Central Apennines^30^.

## Data Availability

The dataset generated during and/or analyzed during the current study are available from the corresponding author on request.
